# Testosterone Reduces Spinal Cord Injury-Induced Effects on Male Reproduction by Preventing CADM1 Defect

**DOI:** 10.22074/cellj.2018.5003

**Published:** 2018-03-18

**Authors:** Hamid Choobineh, Mahsa Kazemi, Mohammad Ali Sadighi Gilani, Tahereh Heydari, Saeed Shokri, Mahshid Bazrafkan, Gholamreza Hassanzadeh

**Affiliations:** 1 School of Allied Medical Sciences, Tehran University of Medical Sciences, Tehran, Iran; 2 Zeoonosis Research Center, Tehran University of Medical Sciences, Tehran, Iran; 3 Department of Anatomy, School of Medicine, Shahid Beheshti University of Medical Sciences, Tehran, Iran; 4 Department of Urology, School of Medicine, Tehran University of Medical Sciences, Tehran, Iran; 5 Department of Biology, Islamic Azad University, Parand Branch, Iran; 6 Department of Anatomy, School of Medicine, Zanjan University of Medical Sciences, Zanjan, Iran; 7 Department of Anatomy, School of Medicine, Tehran University of Medical Sciences, Tehran, Iran

**Keywords:** Cell Adhesion Molecule, Sperm, Spinal Cord Injury, Testis, Testosterone

## Abstract

**Objective:**

This study evaluated the effects of exogenous testosterone molecule-1 (CADM1) pathological defect during early and chronic periods of spinal cord injury (SCI).

**Materials and Methods:**

In this experimental study, testosterone was administered immediately or after one week of SCI induction. Along with quantification of CADM1 gene expression and its immunoreactivity, we evaluated sperm parameters and serum testosterone level post-SCI.

**Results:**

Different grades of abnormalities in sperm parameters and testis architecture were observed along with significant reductions in the level of CADM1 expression and its immunoreactivity in the seminiferous tubules of both acute and chronic SCI groups. Exogenous testosterone, by compensating the serum testosterone level. reduced the percentage of apoptotic and both short head and abnormal sperm froms in the caudal epididymis. Importantly, the beneficial effects of immediate administration of testosterone were prominent. Increases in the level of CADM1 transcription and its immunoreactivity in the testis of SCI mice treated with testosterone were accompanied by improvement of sperm motility as well as testicular Johnsen’s and Miller’s criteria.

**Conclusion:**

Since immediate testosterone treatment improved the immunoreactivity and transcription level of CADM1, the observed beneficial effect of exogenouse testosterone can be attributed to its effect on CADM1 dynamics.

## Introduction

Male infertility due to spinal cord injury (SCI), is associated with a unique semen profile. It is characterized by normal sperm concentrations, low sperm motility, low sperm viability, variable sperm morphology, and abnormal seminal plasma constituents (1-5). In mice with surgically induced SCI, semen quality deteriorates approximately one week post-injury (1, 2). The pathology of asthenozoospermia seems to be multifactorial. The hypothalamic-pituitary-testicular axis dysfunction seen by the third day post-SCI, accounts for the acute effects of SCI on spermatogenesis (1).

During the chronic phase of SCI, abnormal spermatogenesis and/or regression of the seminiferous epithelium is caused through nonendocrine mechanisms (3, 4). Among non-endocrine mechanisms, the abnormal composition of seminal plasma (5) can adversely impact sperm physiology (6). Alterations in testicular function (7, 8) that include a persistent inflammatory process (9, 10), modulation of Sertoli cell functions (11), and impairment of the bloodtestis- barrier (BTB) partially account for spermatogenic impairment in chronic SCI (9). Prevention of some of these abnormalities during early phase of SCI and maintenance of qualitatively complete spermatogenesis during the chronic phase of SCI, have been investigated by different studies. 

The beneficial effects of exogenous testosterone demonstrate that spermatogenic effects of SCI are probably androgen-dependent (2, 12). Huang et al. (12) have shown that altered responsiveness of Sertoli cell mRNA transcripts to exogenous testosterone, changes the endocrine and/or paracrine microenvironment within the seminiferous epithelium and tampers with proliferation and/or differentiation of spermatogenic cells in SCI. Futhermore, the effect of exogenous testosterone on the expression of spermatid-specific proteins such as cAMP responsive element modulator, suggests that abnormal spermiogenesis may also be involved in SCI-induced sperm function impairments (12, 13). The mechanisms underlying the beneficial effects of exogenous testosterone on spermatogenesis of mice with SCI, remain to be determined. 

The direct interaction between spermatogenic and Sertoli cells plays a key role in the regulation of spermatogenesis (14). Cell adhesion molecule-1 (CADM1), a Ca^2+^-independent immunoglobulin-like molecule homophilically and heterophilically interacts with other CADM1 or other protein families (15). 

CADM1 on spermatogenic cells causes heterophilic binding to Sertoli cells and plays an indispensable role in spermatogenesis (14). The expression of CADM1 is detectable from intermediate spermatogonia to early pachytene spermatocytes as well as in step 7 and later spermatids (14, 16). No specific site has been detected as a CADM1 definite morphological structure for spermatogenic and Sertoli cells interaction (14).

CADM1-deficient mice have some similarities to SCI mice in terms of sperm parameters (16, 17) according to the disruption or loss of normal contact between developing sperm cells and Sertoli cells (18). Since testosterone plays an important role in adhesion at the Sertoli-germ cell interface and in regulation of BTB integrity (16), here, we attempted to ascertain the role of CADM1 in SCI pathology and the possible role of exogenous testosterone in its regulation. 

## Materials and Methods

This experimental study was carried out in strict accordance with national guidelines and protocols, and approved by the Institutional Animal Ethical Committee (IAEC no. 03/028/07). All experimental protocols were approved by the Animal Care and Use Committee of Tehran University of Medical Sciences, Tehran, Iran. Healthy adult male albino Balb/c mice (20-25 g and 8 weeks old) were randomly selected. The animals were housed under environmentally controlled conditions with 12/12 hours light/dark cycles. The mice had free access to a standard laboratory diet and clean drinking water ad libitum.

Mice were randomly assigned to two groups underging either SCI or a sham operation. The sham operation included a laminectomy without cord compression. Control animals did not undergo any operation. The control, sham and SCI animals were randomly divided into two categories based on the treatment period lenght.

The first category included five groups (8 animals in each) as follows: i. Animals that were killed 7 days after SCI (SCI7), ii. Animals that received testosterone (Sigma Chem. Co., Germany, Cat. No. T1500) for 7 days immediately after SCI and were killed 24 hours after the last testosterone injection on day 7 post-injury (SCIT7), iii. Animals that received testosterone one week after SCI and were killed 24 hours after last testosterone injection on day day 14 post-injury (SCI-T7), iv. Animals that underwent laminectomy with no cord compression and were killed 7 days after laminectomy (Sham7), and v. Intact animals that underwent no laminectomy (control). The second category included five groups (8 animals in each) as follow: i. Animals that were killed 35 days after SCI (SCI35), ii. Animals that received testosterone immediately after SCI for a 35-day period and were killed 24 hours after the last testosterone injection on day 35 post-injury (SCIT35), iii. Animals that received testosterone one week after SCI for a 35-day period and were killed 24 hours after last testosterone injection (SCI7-T35), iv. Animals that underwent laminectomy with no cord compression and were killed 35 days post- injury (Sham35), and v. Intact animals that underwent no surgical intervention (control).

### Spinal cord injury

SCI were induced under sterile conditions. Animals were anesthetized using ketamine (50 mg/kg)/xylazine (5 mg/kg). The compression injury was induced according to the procedure described by Holtz et al. (19). The T9 to T11 vertebrae were exposed. A laminectomy was performed at the T10 level to expose the cord, leaving the dura intact. To stabilize the vertebral column during the compression, we clamped the dorsal process of the T8. Injury was induced by using the blocking weight- technique. In this technique, a 15 g weight was applied on a 3×3 mm plate (for 5 minutes) to a 5×2.2 mm plate.

After the injury, we rinsed the cord with room temperature saline and removed any residual blood. The wound was closed by separately suturing muscles, skin and the fat pad. After the surgery, animals were injected with sterile saline (2 mL, s.c.) then placed in a warming chamber where their body temperatures were maintained at approximately 37°C until they became fully awake. Once awake, animals were placed individually into their home cages. Post-operative care included manual bladder expression 2-3 times per day at 9:00, 17:00 and 22:00. Animals received cefazolin 3.33 mg/kg per day for 7 days or until their bladders self-expressed. 

We objectively assessed the locomotor and reflex scores of the hind limbs on the day of SCI induction by using a modified Tarlov scale. The Tarlov scale scores were as follows: 0: total paraplegia of the hindlimb, 1: shows no spontaneous movement but responds to a hindlimb pinch, 2: has spontaneous movement but is unable to stand, 3: is able to support weight but unable to walk on a broad flat surface, 4: is able to walk on a broad flat surface, 5: is able to walk on a broad flat surface and support weight on a 1.8 cm wide ledge; and 6: is able to walk on a ledge. Each observer scored the right and left limbs independently, then agreed on a single score for each animal (20).

### Administration of exogenous testosterone

SCI mice received a daily intraperitoneal injection (IP) of testosterone (0.5 mg/kg) either immediately or one week after the surgery for a 7- or 35-day period (2, 12, 21).

### Animal sacrifice and laboratory analysis 

Cohorts of mice from the different treatment groups, sham and control were deeply anesthetized using ketamine and xylazine, then killed according to the schedules. The follow-up experiment was performed during the acute (a time point at which impaired sperm motility was not related to the extent of cord injury) and chronic (a time point at which impaired sperm motility was inversely correlated with the extent of injury) phases of SCI. Heart blood was collected for measurement of serum hormones (12). 

Caudal portions of the epididymides were dissected from the testes immediately after sacrificing the animal. The caudal portions were used for sperm collection in order to analyze sperm parameters. We froze one testis from each mouse immediately in a liquid nitrogen tank for evaluation of CADM1 gene expression. After dissecting each testicle and caudal epididymis, whole body perfusion with formalin was performed for fixation. The other testicle of each mouse was processed for histology analysis using hematoxylin and eosin (H&E) staining and immunohistochemistry. 

### Sperm motility and count analysis 

Caudal epididymides were immersed in 1 ml Hams F10. Epididymal sperm were dispersed by puncturing the epididymis with a 19-gauge needle after which the sperms were incubated at 37°C for 10-15 minutes. A drop (50 mL) of sperm suspension from each mouse was placed on a pre-warmed slide and examined. Sperm motility in the suspension was visually monitored in phase-contrast images of 10-20 microscopic fields. The percentages of the motile, progressive, and immotile sperm were expressed as fractions of total counted sperm according to Shokri et al. (22). The epididymal sperm was obtained from caudal part epididymis. The sperm suspension was diluted with saline that contained 0.5% formalin. A total of 10 µl of the diluted specimen was transferred to one of the hemocytometer chambers for examination under a light microscope.

### Sperm morphology analysis

For this analysis, one sperm droplet (10 µl) was placed and pulled across the slide for preparing the sperm smear. The slide was allowed to dry at room temperature. Staining was performed according to the Papanicolaou method (23). A sperm that lacked a tail and sperms with morphologically abnormal heads and tails (at least in 10 fields) were counted at ×400 magnification. The percentages of abnormal sperm were expressed as a fraction of total counted sperm. Totally 400 sperms were evaluated morphologically. The head lengh of sperms were measured on pictures using ImageJ software.

### Sperm DNA fragmentation analysis 

#### TUNEL assay

We evaluated DNA integrity in epididymal sperm on a prepared air-dried smear slide. The slide was prepared by using 10 µL of specimen and fixed overnight in paraformaldehyde (4%). After fixation, the slide was air- dried then stained for the TUNEL assay with the In-Situ Cell Death Detection Kit (Roche Diagnostics GmbH, Germany). The fixed slides were rinsed in phosphate- buffered solution (PBS, pH=7.4).

Permeation was performed using 2% Triton X-100. The terminal deoxynucleotidyl transferase-labeled nucleotide mixture was added to the slide which was subsequently incubated in a humidified chamber at 37°C for 60 minutes in the dark. Slides were rinsed in PBS (3 times 5 minutes each). Later, converter-peroxidase solution (POD) was added on the slides. Next, the slides were incubated in a humidified chamber (at 37°C for 30 minutes), rinsed three times in PBS, and incubated in the presence of 3-3’ diaminobenzidine (DAB) substrate for 10 minutes.

The slides were further rinsed three times with PBS. The number of sperm per animal were examined using bright-field microscopy. The numbers of brown cells (TUNEL positive) were counted and expressed as the percentage of total sperm cells. Both negative (without enzyme terminal transferase) and positive (incubation with 1 U/mL deoxyribonuclease I for 20 minutes at room temperature) controls were performed for each experiment. In each sample, a total of 400 sperms was evaluated.

#### Sperm chromatin structure assay 

Sperm DNA damage in the sample was measured by the sperm chromatin structure assay (SCSA) (24). In brief, the cell suspension (1-2×10^6^) was treated with a low pH (pH=1.2) detergent solution (0.1% Triton X-100, 0.15 mol/l NaCl and 0.08 mol/l HCl for 30 seconds) as an in situ acid-induced denaturation. SCSA incorporates the metachromatic properties of Acridine Orange (AO) to quantitate the shift from green (native, double-stranded DNA) to red (denatured, single- stranded DNA). The fluorescence cells were stained with 6 mg/l purified AO in a phosphate-citrate buffer (pH=6.0). Cells were analyzed using a FACSort flow cytometer (Facscalibur flow cytometer, BD Scanyose). 

Under excitation at 488 nm, the AO that intercalates with double-stranded DNA emits a green fluorescence and AO that associates with single-stranded DNA emits red fluoresce. A total of 5000 events was accumulated for each measurement. Flow cytometric data were analyzed using Cell Quest Pro software.

The proportions of spermatozoa with increased levels of red and green fluorescence were determined by computer gates. DNA fragmentation index (DFI), as an indicator of the extent of DNA denaturation, is the ratio of red to total (red plus green) fluorescence intensity. This value is the level of denatured DNA over total DNA (25). The DFI value was calculated for each sperm sample.

#### Testis histology analysis

Testicular tissue from each animal was processed for routine histology. The paraffin-embedded sections were deparaffinized, rehydrated, and stained with a solution of H&E. Stained testicular sections were assessed for spermatogenesis, number of germinal cell layers and Johnsen’s score. The number of germinal epithelial layers was counted in 100 seminiferous tubules as described by Miller et al. (26). Johnsen’s method (27) applies a score of 1-10 for each tubule cross-section, according to the presence or absence of the main cell types arranged in the order of maturity. 

The scores are defined as follows: 10: complete spermatogenesis and presence of normally organized tubules, 9: numerous spermatozoa are present but germinal epithelium are disorganized, 8: only a few spermatozoa are present in the section, 7: no spermatozoa is found but numerous spermatids are present, 6: only a few spermatids are present, 5: no spermatozoa or spermatids are present, however numerous spermatocytes could be found, 4: only a few spermatocytes are present, 3: only spermatogonia are present, 2: no germ cells are foung and only Sertoli cells are present, 1: no germ cells and no Sertoli cells are present. In each sectioned sample, 100 tubules were evaluated.

#### Testicular TUNEL assay 

For the evaluation of intratubular nuclei apoptotic events (DNA fragmentation), an ApopTag Peroxidase In Situ Apoptosis Kit (TUNEL, Roche, Germany; cat. no. 11585095001) was used according to the manufacturer’s instructions. Briefly, after fixation, tissue sections were permeabilized by treatment with proteinase K (20 mg/ ml) for 10 minutes. Endogenous peroxidase activity was quenched by treatment with 3% (v/v) H_2_O_2_ in PBS for 10 minutes at room temperature, then incubated with the terminal deoxynucleotidyl transferase (TdT) labelling reaction mixture in a humidified chamber for 1 hour at 37°C. 

After washing, the slides were stained with converter-POD at room temperature for 30 minutes. Finally, slides were developed with DAB. Tissue sections were counterstained with Mayer’s hematoxylin solution, washed, dried, and coverslipped. Positive cells that contained fragmented nuclear chromatin exhibited a brown nuclear stain. Apoptotic index-1 (AI-1) was defined as the number of TUNEL-positive apoptotic cells per 100 tubules and AI-2 was calculated as the number of tubules that contained apoptotic cells per 100 tubules. An expert technician blinded to the source of testicular tissue, performed all measurements.

### Cell adhesion molecule-1 immunohistochemistry

In brief, prepared slides were deparaffinized, rehydrated and used to determine CADM1 immunoreactivity. Antigen retrieval was performed in 10 mM sodium citrate and 0.05% Tween 20 at pH=6.0. Sections were incubated for 15 minutes in 0.5% (v/v) H_2_O_2_ diluted in methanol, rinsed in PBS and incubated with a solution of 1% (w/v) bovine serum albumin (BSA) in PBS for 10 minutes to prevent non-specific binding of the primary antibody. The primary antibody (Rabbit anti-mouse-CADM1 polyclonal antibody, Cat. No. ab3910; Abcam, USA) was applied at a dilution of 1:100 in PBS with 1% (v/v) normal bovine serum at 37°C for 1 hour. 

After washing three times in PBS, sections were incubated with secondary antibody (Dako EnVision Kit, Dako) diluted in PBS for 30 minutes, after which they were developed with DAB. Images were digitally captured. Similar light intensity and filter settings were applied for all specimens. A semiquantitative densitometric measurement of staining was performed by ImageJ software.

First, the background reduced and normalized in all taken pictures. By defining overlayers, different colors were separated. Next, after threshold adjustment on the desired color, the photo was converted to black and white to indicate only the immunostained patterns on samples. The converted photos were converted into 16bit pictures and calibrated according to defined Rodbar scales. Finally, equal circles were randomely placed on the defined stained basal and adluminal compartment of tubules. Optical densities were measured in the basal and adluminal part of seminiferous tubules by evaluating the mean gray values. The average of resulted quantities were quantified and expressed as mean ± SEM for each sample.

### Quantitative real-time polymerase chain reaction 

For CADM1 (GeneID: 54725) RNAs extraction, frozen testis tissues were taken from -80oC storage and homogenized in liquid nitrogen. ß-actin gene was used as a reference gene. Total RNAs were extracted by a purified RNA extraction kit (Roch Life Technologies, USA, Cat. No. 11828665001).The RNAintegrity and its concentration were evaluated and quantified by electrophoresis using a 1% agarose gel and spectrophotometry (Nanodrop, MD1000), respectively. Total RNA (1 µg) was used to generate cDNAs (PrimScriptTM, Takara Bio, Inc., Japan, code RR037A) according to the manufacturer’s directions. 

Quantitative real-time polymerase chain reaction (qRT- PCR) was performed in 96-well plates using a QuantiTect SYBR Green RT-PCR Kit (Ex Taq, Takara Bio, Inc., Japan, code RR820A) according to the manufacturer’s protocols. Each 20 µl reaction mixture contained 1 µl cDNA, 10 µl SYBR Premix EX Taq, and 200 nM primers for each gene. The resultant fluorescence was quantified using an iCycler system (Rotor-Gene TM6000 Corbett, Life Science). PCR reactions initiated at 95oC for 1 minute and followed by 40 cycles under the following conditions: 95°C for 15 seconds, 58°C for 20 seconds, and 72°C for 15 seconds. 

A sequence verified PCR product of 111 bp generated using *CADM1*-specific primers:

F: 5´-CAGGCAGACCATTTACTTCAG-3´
R: 5´-CGAGATTGAGACATTCGTCA-3´

ß-actin specific primers were F: 5´-TGGTGCCAAAAGGGTCATC-3´
R: 5´-CTTCCACGATGCCAAAGTTG-3´

Melting curve data were obtained to confirm specificity in amplification of the correct product by analysis at the dissociation stage. The average cycle threshold (CT) was normalized to ß-actin for each gene in each sample by LinReg PCR software. Changes in CT were calculated using REST software.

### Measurement of testosterone levels

Whole blood was collected from anesthetized mice. Sera were isolated by centrifugation at 2000g for 20 minutes. Serum levels of testosterone were measured by Enzyme Linked Immune Sorbent Assay (ELISA) by using a commercial kit (Sigma-Aldrich. Co., Germany, Cat. No. SE120089) and expressed as ng/ml according to the manufacturer’s instructions.

### Statistical analysis 

Data were expressed as mean ± SEM. The one- way ANOVA test was applied to evaluate significant differences among means. When a significant effect was found, Tukey’s post hoc test was performed. All analyses were performed using SPSS version 16. The statistical significance level was P<0.05. 

## Results

### Epididymal sperm parameters

SCI did not result in any significant differences in the sperm concentration amongst experimental groups (Table 1). Also, there was no significant difference in the percentage of normal sperm between control and sham groups on days 7 and 35 post-injury. SCI induction resulted in a significant (P<0.001) reduction in the percentage of normal sperm on days 7 and 35 post-injury. Testosterone administration to both SCIT7 (75.00 ± 3.60, P<0.001) and SCI7-T7 (65.00 ± 3.46, P<0.05) groups resulted in improvement in the percentage of normal sperm compared to the untreated SCI group (56.00 ± 3.60). On the other hand, prolonged testosterone administration (for 35 days) immediately (83.00 ± 2.08, P<0.001) or one week later (60.33 ± 1.20, P<0.05) after SCI, resulted in a significant increase in the percentage of sperm with normal morphology compared to the untreated SCI group (45.33 ± 3.18). 

**Fig.1:**
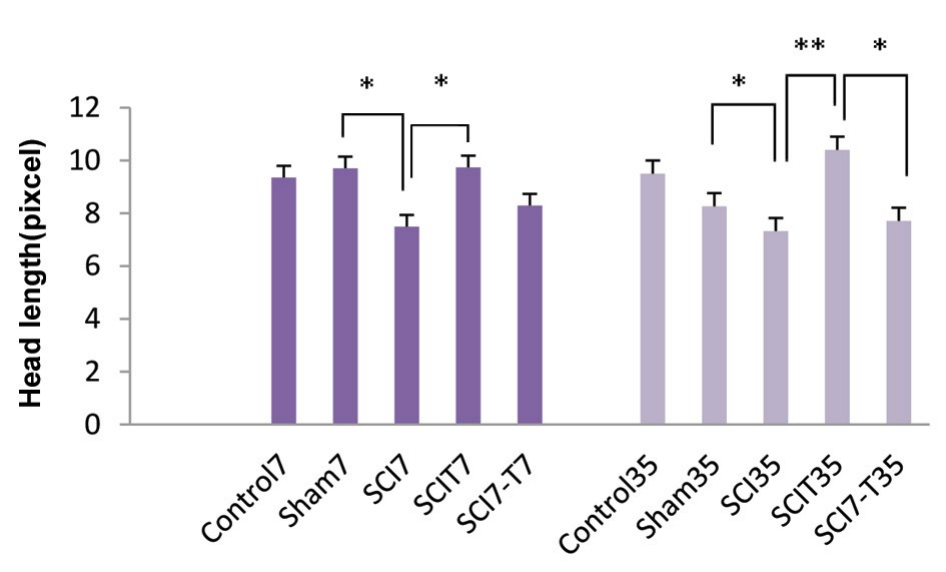
Effect of spinal cord injury (SCI) and different patterns of testosterone administration duringacute and chronic phases post-injury on the head length of sperm obtained from the caudal part of the epididymis. Control: Intact animals, Sham7 and sham35: Mice with laminectomy without SCI, SCI7 and SCI35: Mice with SCI that were killed 7 and 35 days post-injury, SCIT7 and SCIT35: Mice that received testosterone for 7 and 35 days immediately after SCI and were killed 24 hours after the last testosterone injection on day 8 and 36 post-injury, SCI7-T7 and SCI7-T35: Mice that received testosterone beginning one week after SCI and were killed 24 hours after the last testosterone injection on day 14 and 42 post-injury. *; P<0.01 and **; P<0.001.

The length of sperm head reduced significantly (P<0.01) in both acute (7.49 ± 0.57) and chronic (7.32 ± 0.5) phases of SCI compared to the sham7 (9.7 ± 0.11) and sham35 (8.26 ± 1.15) groups. Testosterone treatment immediately post-SCI caused a significant increase in the head length of sperm in both acute (P<0.01) and chronic (P<0.001) phases (Fig .1). Laminectomy did not change the head length of sperm compared to the control group. Testosterone treatment one week later of SCI induction did not increase sperm head length in either acute or chronic phases.

**Fig.2:**
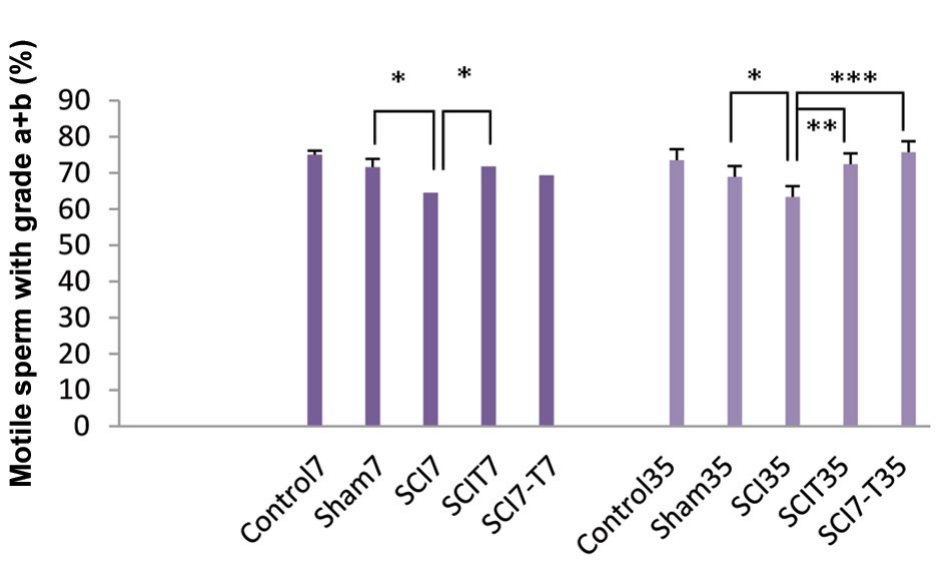
Effect of spinal cord injury (SCI) and different patterns of testosterone administration during acute and chronic phases post-injury on sperm motility. Control: Intact animals, Sham7 and sham35: Mice with laminectomy without SCI, SCI7 and SCI35: Mice with SCI that were killed 7 and 35 days post-injury, SCIT7 and SCIT35: Mice that received testosterone for 7 and 35 days immediately after SCI and were killed 24 hours after the last testosterone injection on day 8 and 36 post-injury, SCI7-T7 and SCI7-T35: Mice that received testosterone beginning one week after SCI and were killed 24 hours after the last testosterone injection on day 14 and 42 post-injury. *; P<0.05, **; P<0.01, and ***; P<0.001.

Sperm motility was affected in the experimental groups (Fig .2). Sperm motility significantly (P<0.01) reduced in both acute (SCI7: 64.49 ± 2.30, sham7: 71.55 ± 3.61) and chronic (SCI35: 63.32 ± 1.73, sham35: 68.88 ± 3.34) groups versus their dedicated sham groups. Exogenous testosterone resulted in a significant increase in sperm motility parameters in SCIT7, SCIT35 and SCI7-T35 groups. There were no significant differences in the sperm parameters between control animals and the sham group . We did not detect any significant differences in the efficiency of testosterone between immediate and time lapse administration post-SCI. 

**Table 1:** Effect of acute and chronic spinal cord injury (SCI) and exogenous testosterone treatment on sperm count, the percentage of normal morphology, TUNEL positive and sperm chromatin structure assay (SCSA) positive sperm


Group	Count (×10^6^)	Normal (%)	TUNEL positive cells (%)	DFI (%)

Control7	2.88 ± 0.05	90.00 ± 2.51	10.16 ± 0.44	8.26 ± 0.53
Sham7	2.94 ± 0.06	84.67 ± 4.41	9.5 ± 0.57	7.5 ± 0.28
SCI7	2.89 ± 0.02	56.00 ± 3.60^2^	23 ± 0.50^3^	18 ± 0.55^3^
SCIT7	2.99 ± 0.06	75.00 ± 3.60^2^	19 ± 1.0^3^	12 ± 1.5
SCI7-T7	2.95 ± 0.01	65.00 ± 3.46^1^	20.5 ± 0.55	15.5 ± 1.33
Control35	2.80 ± 0.02	88.00 ± 1.73	9.3 ± 0.17	8.6 ± 0.37
Sham35	2.97 ± 0.01	79.00 ± 0.57	9.66 ± 0.72	8.16 ± 0.44
SCI35	2.87 ± 0.05	45.33 ± 3.18^2^	60 ± 1.15^3^	26 ± 0.50^3^
SCIT35	3.13 ± 0.03	83.00 ± 2.08^2^	16 ± 1.50^3^	12 ± 1.15^3*^
SCI7-T35	2.94 ± 0.09	60.33 ± 1.20^1^	12 ± 0.55^3^	18 ± 0.57^3*^


Sham groups were compared to the control groups. Both SCI7 and SCI35 were compared to their Sham groups. SCIT7 and SCI7-T7 were compared to SCI7 group. SCIT35 and SCI7-T35 were compared to SCI35 group. Control; Intact animals, Sham7 and sham35; Mice with laminectomy without SCI, SCI7 and SCI35; Mice with SCI that were killed 7 and 35 days post-injury, SCIT7 and SCIT35; Mice that received testosterone for 7 and 35 days immediately after SCI and were killed 24 hours after the last testosterone injection on day 8 and 36 post-injury, SCI7-T7 and SCI7-T35; Mice that received testosterone beginning one week after SCI and were killed 24 hours after the last testosterone injection on day 14 and 42 post-injury. ^1^; P<0.05, ^2^; P<0.01, ^3^; P<0.001, and *; Comparison between SCIT35 and SCI7-T35 groups, P<0.01.

### Sperm DNA damage 

The percentage of TUNEL positive cells and DFI showed no significant differences between sham groups compared to the control groups (Table 1). The TUNEL and SCSA assays had the same results amongst the SCI groups. Both acute and chronic phases of SCI were accompanied by significant increases (P<0.001) in the percentage of TUNEL positive sperm in SCI7 group (16.5 ± 0.50) versus sham7 group (9.30 ± 0.57) as well as in SCI35 group (60 ± 1.15) versus sham35 group (9.66 ± 0.72). DFI significantly increased in SCI7 group (18 ± 0.55) comared to sham7 group (7.5 ± 0.28) and in SCI35 group (26 ± 0.50) compared to sham35 group (8.16 ± 0.44). Interestingly, exogenous testosterone significantly reduced the percentage of TUNEL positive sperm only when administered immediately to the SCIT7 group. The percentage of TUNEL positive cells and DFI showed significant reduction (P<0.001) in SCIT35 and SCI7-T35 groups compared to SCI35 group.

### Serum testosterone fluctuations 

**Fig.3:**
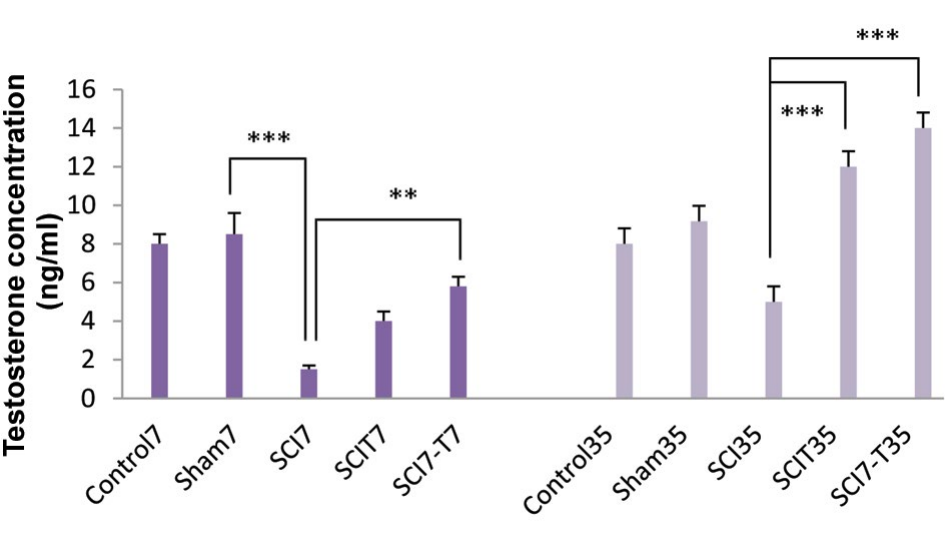
Effect of spinal cord injury (SCI) and different patterns of testosterone administration during acute and chronic phases post- injury on serum testosterone levels. Control: Intact animals, Sham7 and sham35: Mice with laminectomy without SCI, SCI7 and SCI35: Mice with SCI that were killed 7 and 35 days post-injury, SCIT7 and SCIT35: Mice that received testosterone for 7 and 35 days immediately after SCI and were killed 24 hours after the last testosterone injection on day 8 and 36 post- injury, SCI7-T7 and SCI7-T35: Mice that received testosterone beginning one week after SCI and were killed 24 hours after the last testosterone injection on day 14 and 42 post-injury. **; P<0.01 and ***; P<0.001.

Serum testosterone concentration was quantified inall experimental groups. There were no significantdifferences between the sham and control groups. Seven days post SCI, the level of serum testosteronereduced significantly (P<0.001) in SCI7 group incomparison to sham7 group (Fig .3). Interestingly, thechronic phase of SCI (35 days after injury) did not show a significant reduction in testosterone level.

Exogenous testosterone administered immediately post-SCI non-significantly increased the testosterone level compared to the untreated SCI7 group. There was an increase in the level of serum testosterone in SCI7-T7 group (5.8 ± 0.57) compared to SCI7 group (1.5 ± 0.28, P<0.01). During the chronic phase of SCI, testosterone administration to SCIT35 (12 ± 1.15) and SCI7-T35 (14 ± 1.53) groups significantly increased serum testosterone levels (P<0.001) compared to SCI35 group (9.17 ± 0.91). 

### Testicular parameters 

The effects of SCI and exogenous testosterone treatment on germinal epithelium were quantified according to the Miller’s and Johnsen’s criteria. The percentages of apoptotic cells/tubules per 100 evaluated seminiferous tubules, were reported as the apoptotic indices (Table 2).

Epithelium parameters that included diagonal and thicknessmeasurements of the seminiferous tubules, apoptotic indices, and Miller’s and Johnsen’s criteria showed no significant differences between control and sham groups. Seven days post SCI induction, the thickness and diameter of tubules showed a significant reduction. There were also reductions in Miller’s and Johnsen’s criteria but an increase in TUNEL indices. Although the same pattern of fluctuations was observed in TUNEL indices and both Miller’s and Johnsen’s criteria, there were no significant differences in the thickness and diameter of tubules of SCI-35 and sham35 groups. Immediate testestrone treatment in both SCI7 and SCI35 decreased the level of TUNEL indices. 

There was also significant improvement in Miller’s and Johnsen’s criteria following immediate testosterone administration. Although the significant beneficial effects of testosterone administration after one week of SCI induction were observed in SCI-T7 and SCI-T35 groups, its ameliorating effects were observed to a lesser extent compared to those groups with immediate testosterone administration. 

### Transcription and immunoreactivity of CADM1 

**Fig.4:**
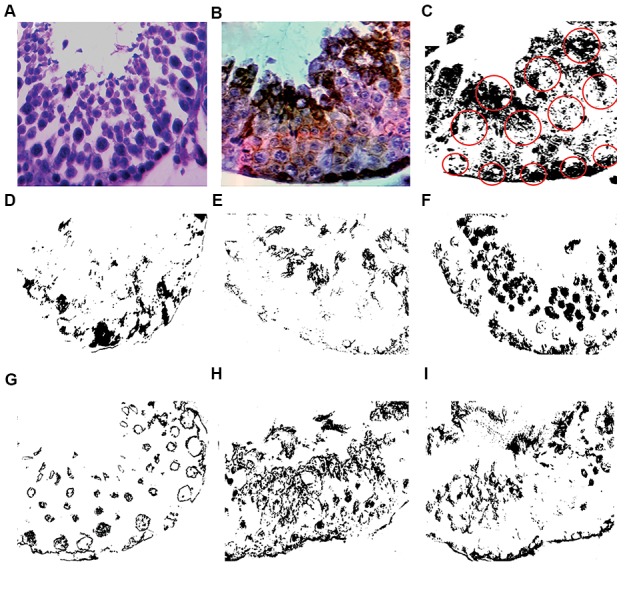
Effect of spinal cord injury (SCI) and testosterone treatment on the descriptive feature of cell adhesion molecule-1 (CADM1). The patterns of immunoreactivityin the experimental groups. A. Represents the negative control, B. Control animal without spinal cord injury (SCI). Brownish parts in the picture B indicate thepositive immunoreactivity of CADM1 in different cellular layers of seminiferous tubule, C. Represents the same converted picture of B used in densitometry procedure, D. Immunoreactivity pattern in the SCI7 group, E. Immunoreactivity pattern in the SCI35 group, F. Immunoreactivity pattern in the SCIT7 group, G. Immunoreactivity pattern in the SCI7-T7 group, H. Immunoreactivity pattern in the SCIT35, and I. Immunoreactivity pattern in SCI7-T35.

CADM1 belongs to the spermatogenic immunoglobulin superfamily. CADM1 immunoreactivity results in specific localization of the receptor in testicular germ cells. Similar to previous studies (28, 29), we detected CADM1 immunoreactivity on the cell surface of intermediate spermatogonia. Even with regression of the seminiferous epithelium, immunoreactivity was detectable around the spermatogonia. We quantified optical density (OD) of CADM1 immunoreactivity in the basal and adluminal compartments of the germinal epithelium (Table 3, Fig .4).

There were no significant differences in OD of these compartments between control and sham groups. Acute and chronic SCI groups showed a profound reduction in OD of the basal compartment in SCI7 (0.001 ± 0.00) versus sham7 (0.004 ± 0.00) groups and in SCI35 (0.001 ± 0.00) versus sham35 (0.004 ± 0.00) groups. In the adluminal compartment, the OD profoundly reduced in SCI7 (0.00 ± 0.00) versus sham7 (0.008 ± 0.00) groups and SCI35 (0.001 ± 0.00) versus sham35 (0.008 ± 0.00) groups. Interestingly, immediate testosterone treatment significantly prevented profound reduction of CADM1 immunoreactivity in both basal and adluminal compartments of the seminiferous epithelium. On the other hand, time lapse testosterone treatment did not prevent a reduction in receptor immunoreactivity in both acute and chronic phases of SCI. 

The changes in transcription of the CADM1 receptor gene after SCI induction and testosterone treatment were evaluated quantitatively (Table 3). There were no significant differences in the transcription levels of basal and adluminal compartments of the epithelium between control and sham groups. SCI caused a significant reduction in the transcription level of the receptor in SCI7 (0.13 ± 0.0) versus sham7 (1.75 ± 0.0) groups and in SCI35 (0.17 ± 0.0) versus sham35 (1.67 ± 0.01) groups. Of note, the same pattern of reduction was seen in the chronic phase. Both immediate and time lapse testosterone administration resulted in a significant (P<0.001) increase in the level of receptor transcription during the acute and chronic phases of SCI. 

**Table 2:** Effect of spinal cord injury (SCI) and testosterone treatment on germinal epithelium


Group	TUNEL	Miller	Johnsen	Thickness	Diameter(mm)
	AI-I	AI-II					

Control7	23 ± 2.5		14.33 ± 0.88	4.94 ± 0.23	8.83 ± 0.16	15.5 ± 1	46.62 ± 1.11
Sham7	25 ± 3.51		15 ± 2.51	5.26 ± 0.17	9.36 ± 0.18	15.53 ± 0.6	44 ± 2.30
SCI7	194 ± 8.71^3^		70 ± 3.21^3^	3.38 ± 0.31^3^	4.83 ± 0.15^3^	11.9 ± 0.57^3^	32.36 ± 0.88^3^
SCIT7	36.33 ± 2.72^3^		25.33 ± 0.88^3#^	4.65 ± 0.37^1*^	7.80 ± 0.15^3€^	15.74 ± 0.57^1^	45.1 ± 2.88^3^
SCI7-T7	47.67 ± 1.45^3^		39 ± 2.64^3#^	3.38 ± 0.13^*^	5.33 ± 0.33^€^	17.3 ± 1.15^2^	51.6 ± 0.57^3^
Control35	24.67 ± 1.85		14.2 ± 1.20	5.28 ± 0.13	9.51 ± 0.04	15.5 ± 0.57	46.73 ± 0.57
Sham35	23 ± 0.8		14.67 ± 1.45	4.66 ± 0.33	8.5 ± 0.28	13.86 ± 0.31	44.66 ± 0.88
SCI35	91.67 ± 5.60^3^		61.67 ± 2.02^3^	2.70 ± 0.15^2^	5.42 ± 0.22^3^	16.77 ± 0.57	47.79 ± 0.57
SCIT35	37.67 ± 1.45^3^		28 ± 0.57^3##^	4.68 ± 0.17^1^	7.74 ± 0.16^2**^	14.3 ± 0.57	41.52 ± 1.15
SCI7-T35	48.67 ± 1.20^3^		57.33 ± 1.76^##^	3.70 ± 0.15	6.55 ± 0.29^1**^	13.95 ± 1	40.75 ± 1.73


SCI7 and 35 were compared to the sham groups. SCIT7 and SCI7-T7 were compared to the SCI7 group. SCIT35 and SCI7-T35 were compared to the SCI35 group. Control; Intact animals, Sham7 and sham35; Mice with laminectomy without SCI, SCI7 and SCI35; Mice with SCI that were killed 7 and 35 days post-injury, SCIT7 and SCIT35; Mice that received testosterone for 7 and 35 days immediately after SCI and were killed 24 hours after the last testosterone injection on day 8 and 36 post-injury, SCI7-T7 and SCI7-T35; Mice that received testosterone beginning one week after SCI and were killed 24 hours after the last testosterone injection on day 14 and 42 post-injury. ^1^; P<0.05, ^2^; P<0.01 and ^3^; P<0.001, *; Comparison between SCIT7 and SCI7-T7 groups, P<0.05, **; Comparison between SCIT35 and SCI7-T35 groups, P<0.05, #; Comparison between SCIT7 and SCI7-T7 groups, P<0.01, ##; Comparison between SCIT35 and SCI7-T35 groups, P<0.01, and €; Comparison between SCIT7 and SCI7-T7 groups, P<0.001

**Table 3:** Effect of spinal cord injury (SCI) and testosterone treatment on the expression and transcription level of cell adhesion molecule-1 (CADM1)


Group		CADM1	
	Germinal epithelium immunoreactivity optical density (OD)		Quantitative real-time PCR
	Basal	Adluminal	

Control7	0.0042 ± 0.00	0.0083 ± 0.00	1.67 ± 0.08
Sham7	0.004 ± 0.00	0.008 ± 0.00	1.75 ± 0.0
SCI7	0.001 ± 0.00^3^	0.00 ± 0.00^3^	0.13 ± 0.0^3^
SCIT7	0.003 ± 0.00^3#^	0.004 ± 0.00^3*^	0.8 ± 0.0^3€^
SCI7-T7	0.001 ± 0.00^#^	0.001 ± 0.00^*^	0.45 ± 0.01^3€^
Control35	0.004 ± 0.00	0.0081 ± 0.00	1.78 ± 0.00
Sham35	0.004 ± 0.00	0.008 ± 0.00	1.67 ± 0.01
SCI35	0.001 ± 0.00^3^	0.001 ± 0.00^3^	0.17 ± 0.0^3^
SCIT35	0.002 ± 0.00^2^	0.005 ± 0.00^3##^	0.89 ± 0.02^3**^
SCI7-T35	0.001 ± 0.00	0.001 ± 0.00^##^	0.3 ± 0.0^3**^


SCI7 and 35 were compared to the sham groups. SCIT7 and SCI7-T7 were compared to the SCI7 group. SCIT35 and SCI7-T35 were compared to the SCI35 group. Control; Intact animals, Sham7 and sham35; Mice with laminectomy without SCI, SCI7 and SCI35; Mice with SCI that were killed 7 and 35 days post-injury, SCIT7 and SCIT35; Mice that received testosterone for 7 and 35 days immediately after SCI and were killed 24 hours after the last testosterone injection on day 8 and 36 post-injury, SCI7-T7 and SCI7-T35; Mice that received testosterone beginning one week after SCI and were killed 24 hours after the last testosterone injection on day 14 and 42 post-injury. ^1^; P<0.05, ^2^; P<0.01, ^3^; P<0.001, *; Comparison between SCIT7 and SCI7-T7 groups, P<0.01, #; Comparison between SCIT7 and SCI7-T7 groups, P<0.001, €; Comparison between SCIT7 and SCI7-T7 groups, P<0.001, ##; Comparison between SCIT35 and SCI7-T35 groups, P<0.001, and **; Comparison between SCIT35 and SCI7-T35 groups, P<0.001.

## Discussion

Although impairment of spermatogenesis in SCI is well documented, little is known about its underlying causes. Defects in Sertoli-germ cell interactions after SCI can result in defects of sperm function and impairment of spermatogenesis (12, 13). CADM1, a member of the immunoglobulin super family of spermatogenic cells, has been shown to play an indispensable role in spermatogenesis by forming heterophilic bonds with Sertoli cells (30, 31). In order to determine the role of CADM1, we evaluated the defects in sperm, testis architecrure, and CADM1 transcription and expression after SCI 12, 13 as well as the beneficial effects of sub-acute and chronic exogenous testosterone treatment on the aforementioned parameters in mice with SCI.

The causes of post-SCI asthenozoospermia, as we observed in both SCI7 and SCI35 groups, can be categorized into two groups: acute and chronic events. Multiple steps in spermatogenesis, sperm maturation, or both may be affected by endocrine and neural-related mechanisms of SCI (1, 3, 4). Hormone deficiency temporarily accounts for an early predominant cause of infertility in SCI (5, 9). In accordance with previous studies, withdrawal of testosterone 7 days post-SCI as a condition similar to the acute phase of SCI, has resulted in destruction of testicular architecture and retrived epididymal sperm, seven days post-SCI.

Similar to previous reports (5), in parallel with increasing apoptotic indices, both diameter and thickness of tubules along with Johnsen´s and Miller´s criteria reduced in testis 7 days post-SCI. Despite no fluctuations in sperm concentration, variable abnormal sperm morphology (1, 12), and low sperm motility with a concomitant increase in sperm DNA fragmentation (32-34) were observed in SCI7 group. 

Actually, even with maintenance of complete spermatogenesis in mice with chronic SCI, gradual disappearance of proliferating spermatogonia and eventual regression of the seminiferous epithelium were apparent in seminiferous epithelium (5, 8, 15) which was similar to humans (9). It was clearly indicated that normal hormonal milieu is almost restored by 14 days after SCI (5, 9). Based on serum testosterone level of SCI35 group, the relatively normal function of the pituitary-testis hormone axis was restored in the chronic phase of SCI. By the way, sperm motility, morphology, and DNA integrity continue to deteriorate during this phase (2-5).

The increased number of sperm with shorter head lenght in both SCI7 and SCI35 groups, pave the way to consider the crosstalk of sperm and Sertoli cells following SCI induction. Testosterone is essential for differentiation of round spermatids into elongated spermatids at stages VII-VIII. Withdrawal of testosterone during the acute phase affects adhesion between round spermatids and Sertoli cells and impairs both the development of germ cells, particularly round spermatids, and their attachment to the germinal epithelium. Previousely, it was shown that CADM1-deficient mice have lower percentages of elongated spermatids but increased percentages of round spermatids (17). 

Since this phenomenon was also observed in SCI mice in the current study, we proposed that it may indicate failure of round spermatids development into elongating spermatids, according to the disruption or loss of normal contact with Sertoli cells (16). Possible sloughing of round spermatids from the epithelium during the acute phase of SCI (17) could be related to a defect in CADM1. Transcription of CADM1 terminates in the early spermatocytes; however, translation of remaining mRNA of receptor restarts in the round spermatids at step VII and later. 

The mRNA is probably stored as a ribonucleoprotein complex in the cytoplasm. Several days later, it is recruited to the translation machinery (35-37). Interestingly, the quantity of CADM1 expression in the seminiferous tubules showed significant reduction, 7 days post-SCI. In parallel, a significant reduction in the level of CADM1 immunohistochemistry was observed in both basal and adluminal compartments of tubuls. The presence of sperm with shorter head lenght in the chronic phase of SCI, can be mainly attributed to nonendocrine mechanisms that mediated the effects of SCI on spermatogenesis (35). 

To emphasize the role of CADM1, it is worth to mention that dysfunction of germ cells and spermatogenesis, defect in the production of mature sperm cells, low sperm number, low motility, and abnormal sperm morphology were concomitantly observed in the testes of CADM1-deficient mice (25). According to our observations, the reported delayed maturation from spermatocytes to spermatids, sloughing of spermatids from seminiferous epithelia into the lumen, apoptosis and arrest of spermatid maturation (36), in CADM1-deficient mice were common in SCI-injured animals as well. Accordingly, we observed a significant reduction in the transcription of CADM1 in the chronic phases of SCI. 

Since there were no significant differences in sperm counts between SCI and sham groups, the observed reduction in qRT-PCR results indicated a net reduction in CADM1 transcription. In parallel, densitometry quantification showed that CADM1 expression in basal or adluminal compartments of seminiferous tubules significantly reduced in chronic phase post- SCI which was in harmony with the acute phase. 

It has been reported that testosterone alone is sufficient to restore and maintain complete spermatogenesis in hypophysectomized rats (6). Previously, the beneficial effects of exogenous testosterone have been demonstrated in SCI rats (2) and Sertoli cells (11). As is shown in Figure 3, despite insignificant hormonal increase in SCIT7 group, exogenouse testosterone administration could increase the hormonal level in SCI-T7, SCI35 and SCI7-T35 groups. It has been shown that testosterone implantation results in dose- dependent increases of serum testosterone levels in SCI-injured animals (12). The beneficial effects of testosterone compensation on testicular architecture and sperm parameters were not homogenouse in groups with immediate treatment and in groups with one week interval.

Specifically, similar to previous reports (1, 12), immediate testosterone administration improved both the testicular parameters (increases in the Johnsen’s and Miller’s criteria, while reductions in the testicular apoptotic indices) and sperm parameters (increases in the percentage of motile sperm and normal morphology but reductions in short head sperms) in SCIT7 and SCI35 groups. To a lesser extent, the beneficial effects of exogenouse testosterone on the testicular parameters (increases in the Johnsen’s and Miller’s criteria but reductions in the testicular apoptotic indices) and sperm parameters (increases in the percentage of sperm with normal morphology but reductions in the short head sperms) were observed in SCI7-T7 and SCI7-T35 groups.

It is worth to consider that despite compensation of serum testosterone by exogenouse administration of testosterone to SCI7-T7 group, there was a lack of reduction in the percentage of TUNEL positive sperm and DFI. In parallel, we did not observe motility improvement in this group. The lack of reduction in the percentage of TUNEL positive sperm and DFI was also observed in SCIT7 group. For explaining this discrepancy, it should be mentioned that no consistent correlation between hormone abnormalities and semen quality has been determined (37).

Hence, it was possible that SCI, by altering epididymal autonomic innervation might affect sperm movement in the cauda epididymis during sub-acute phase of SCI (20). Although this might not affect intrinsic motility of the sperm, it might impact final mature sperm movement and its apoptotic status (5). Moreover, it was shown that early adverse effects of SCI on sperm motility exerted by seminal plasma are mediated through inducing a mitochondrial dysfunction. Glycolysis and mitochondrial respiration blockage, by inducing oxidative and apoptotic events, account for later consequences in sperm motility and vitality (10).

It was previousely shown that although exogenous testosterone maintains sperm viability and mitochondrial potential in SCI rats, it cannot improve sperm motility (12). Apart from this, we observed that the compensatory level of testosterone can significantly reduce short head sperms in the retrived samples of both SCIT7 and SCIT35 groups. The weaker ameliorating effects of exogenouse testosterone in the groups with one-week hormonal depriviation suggest that multiple mechanisms are involved in the effects of exogenous testosterone. 

It has been speculated that poor sperm parameters (e.g. DNA damage andlow motility) in sperm retrieved from the cauda epididymis might be attributed to alterations in testicular function as well (7, 8). Lack of physical or biochemical support for spermatogenic cells has been shown to result in loss of normal Sertoli cell function (1) and abortive apoptosis during spermatogenesis (38). The observed improvement in the testicular architecture by immediate testosterone administration, may suggest that testosterone effects are partially mediated by testis environment. 

Interestingly, the expression and transcription of CADM1 changed in both acute and chronic SCI groups. We quantified its expression and transcription in testosterone-treated groups during the acute and chronic phases of SCI. Although the increased level of CADM1 gene transcription and expression were observed in SCI7-T7 and SCI7-T35 groups, those groups that received immediate testosterone treatment (SCIT7 and SCIT35) showed more marked increases in CADM1 transcription level as well as the quantity of CADM1 immunoreactivity in basal and adluminal compartments of tubules. Testosterone promotes adhesion at the Sertoli-Sertoli and Sertoli-germ cell interface and is vital for cell adhesion (39). Moreover, CADM1 affects specific molecules directly involved in sperm motility which can affect asthenozoospermia (40). CADM1 is also responsible for the attachment of germ cells to the seminiferous epithelium and spermatid morphogenesis in the testis (23). Since hormones can regulate the cross-talk between signaling molecules and different regulatory levels of cell junction proteins processing including transcriptional, posttranscriptional and post-translational modifications, it is crucial for the precise control of cell junction modulation (22). This finding led us to speculate that immediate exogenous testosterone administration may play a key dominant role in modulating CADM1 dynamics in the testis by balancing cytokines and hormonal cross-talk in the inflammatory environment of both acute and chronic SCI and regulating junction complex integrity and trafficking (13, 22, 25).

## Conclusion

The results of the present study indicated the probable role of CADM1 in the pathology of SCI. In addition to the effect of testosterone withdrawal during the acute phase of SCI on sperm parameters and testis histology, it has been well-defined that despite hormonal compensation during the chronic phase of SCI, the male reproductive system might be affected by non-hormonal causes. In general, various evidence showed that immediate hormonal treatment in acute and chronic phases of SCI was much more efficient than testosterone administration with a 7-day time lapse post-SCI. Specifically, different sperm parameters along with the testis histology and the pattern of CADM1 transcription and expression clarified the beneficial effect of immediate testosterone treatment during the chronic phase of SCI. According to the profound role of testosterone treatment on the immunoreactivity and transcription level of CADM1, additional studies should be conducted to clarify the exact role of testosterone in CADM1 trafficking. 
